# Anti-inflammatory effect of *Prunus yedoensis* through inhibition of nuclear factor-κB in macrophages

**DOI:** 10.1186/1472-6882-13-92

**Published:** 2013-04-30

**Authors:** Juyeong Lee, Gabsik Yang, Kyungjin Lee, Mi-Hwa Lee, Ji-Whan Eom, Inhye Ham, Ho-Young Choi

**Affiliations:** 1 Department of Herbology, College of Oriental Medicine, Kyung Hee University, 1 Hoegi-Dong, Dongdaemun-Gu, Seoul, Republic of Korea; 2 Department of Physiology, College of Oriental Medicine, Sangji University, Wonju-si Gangwon-do 220-702, Republic of Korea; 3 College of Oriental Medicine, Institute of Oriental Medicine, Kyung Hee University, 1 Hoegi-Dong, Dongdaemun-Gu, Seoul, Republic of Korea

**Keywords:** *Prunus yedoensis*, Inducible nitric oxide synthase, Cyclooxygenase-2, Nuclear factor-κB

## Abstract

**Background:**

*Prunus yedoensis* (PY) is a traditional anti-allergy and anti-inflammatory herb medicine used in South Korea. However, until date, little is known regarding its mechanism of action.

**Methods:**

In order to elucidate the mechanism of anti-inflammatory effect of PY, the constituents of PY were analysed by high performance liquid chromatography (HPLC), and nitric oxide (NO) and prostaglandin E_2_ (PGE_2_) production were measured enzyme-linked immuno sorbent assay (ELISA). The expression levels of inducible nitric oxide synthase (iNOS), cyclooxygenase-2 (COX-2), and nuclear factor-κB (NF-κB) were also measured by western blotting in lipopolysaccharide (LPS)-induced RAW 264.7 macrophage cells treated with PY.

**Results:**

The results indicate that (50, 100 μg/mL) methanol and ethyl acetate fractionation extracts of PY not only inhibited LPS-mediated NO production and iNOS expression, but also decreased LPS-induced PGE_2_ production and COX-2 expression. The anti-inflammatory effects of PY were also due to the attenuation of nuclear translocation of NF-κB, as evaluated by the use of anti-p50 on nuclear fractions. LPS-induced nuclear translocation of NF-κB decreased significantly by the methanol extract and ethyl acetate fraction of PY. High performance liquid chromatography (HPLC) analyses revealed that methanol extract and ethyl acetate fraction have similar patterns of retention time and peaks.

**Conclusion:**

Our results demonstrate that methanol extracts and the ethyl acetate fraction of PY have anti-inflammatory properties, thus emphasizing the potential of PY as a natural health product.

## Background

The inflammatory response in an organism protects the host against tissue injury and microbial invasion. As such this response is short lived, failing which it results in the pathogenesis of many immune related diseases [1]. Chronic (or acute) inflammation constitutes multiple processes, which are activated by inflammatory or immune cells. Of these activated cells, macrophages play a central role in managing many different immune pathological phenomena such as the over-production of pro-inflammatory cytokines and inflammatory mediators [2]. iNOS and COX-2 responsible for the elevated level of NO and prostaglandins, respectively, are well known key pro-inflammatory mediators in many diseases [3,4]. Many studies have shown that the chronic phase of inflammation is closely associated with an increase in iNOS and COX-2 activity [5,6].

NO has been demonstrated to be an important regulatory molecule for diverse physiological functions such as vasodilation, neural communication, and host defence [7,8]. NO is a free radical generated through the conversion of l-arginine to citrulline, catalysed by NO synthase (NOS). NOS in macrophages and hepatocytes is inducible, and its activation is Ca2+ independent. iNOS catalyses the formation and release of a large amount of NO, which then plays a key role in disease pathophysiology [9,10].

COX catalyses the conversion of arachidonic acid to prostaglandin H_2_, the precursor of a variety of biologically active mediators such as PGE_2_, prostacyclin, and thromboxane A2 [11,12]. Two forms of this enzyme have been identified: COX-1, a constitutive cyclooxygenase, and COX-2, which is induced and activated at the site of the inflammation [13-15]. COX-2 is rapidly induced in macrophages and endothelial cells by pro-inflammatory cytokines and maybe responsible for the edema and vasodilation associated with inflammation. Overproduction of inflammatory mediators involves many diseases, such as rheumatoid arthritis, chronic hepatitis, and pulmonary fibrosis [16-18]. Hence, inhibition of the production of these inflammatory mediators may prevent or suppress a variety of inflammatory diseases, including sepsis and endotoxemia [19].

PY is a traditional anti-allergy and anti-inflammatory herb medicine used in South Korea. However, little is known about its mechanism of action and effectiveness as an anti-inflammatory agent. In the present study, we investigated if a methanol and ethyl acetate extract of PY inhibits LPS-induced production of nitric oxide, prostaglandin E_2_, and the expression of iNOS and COX-2 proteins through the inhibition of NF-κB in macrophages.

## Methods

### Plant material

The cortex of *Prunus yedoensis* (PY) was purchased from an oriental drug company, Dongwoodang co., LTD (Yeongchen, Kyeongbuk, Republic of Korea). PY was collected on June, 2007. This plant material was authenticated by Dr. Ho-Young Choi and voucher specimen (No. PY 001) was deposited in the laboratory of herbology, college of Oriental Medicine, Kyung Hee University, Seoul, Korea. The cortex of PY (3 kg) was extracted with 100% MeOH three times for 3 h under heating mantle-reflux. The resultant extract was condensed with rotary vacuum evaporator (N-N series, EYELA, Japan) and partitioned with Chloroform, Ethyl acetate and Water fraction. After each partition, the solutions were filtered and the solvents were evaporated in the rotary vacuum evaporator. The extract yielded Chloroform (3.5 g), Ethyl acetate (40 g) and H_2_0 (36.2 g) soluble extractions.

### Cell culture and sample treatment

The murine macrophage cell line, Raw 264.7, was obtained from the Korea Research Institute of Bioscience and Biotechnology, South Korea. The cells were grown in high glucose DMEM Medium (Hyclone Road Logan, USA) containing 10% fetal bovine serum and 10 ml/L anti-biotics. Cells were incubated in humidified 5% CO_2_ atmosphere at 37°C. Cells were incubated with the tested samples at increasing concentrations (50 or 100 μg/ml) or positive chemical for 1 h and then induced with LPS (10 μg/ml) for the indicated time.

### MTS-tetrazolium salt assay

The Promega CellTiter 96® AQ_ueous_ Non-radioactive Cell Proliferation Assay was used to measure the cytotoxicity of test gases based on numbers of viable cells in culture (Promega, 2001). The MTS (3-(4,5-dimethylthiazol-2-yl)-5-(3-carboxymethoxyphenyl)-2-(4-sulfophenyl)-2H- tetrazolium) assay is based on the ability of viable cells to convert soluble tetrazolium salt to a formazan product. After adding MTS/PMS reagent cell cultures were incubated at 37°C for 1 h, and optical densities were measured using an ELISA plate reader (VersaMax™, Molecular Device, USA) at a wavelength of 490 nm.

### Determinations of nitrite concentrations

The nitrite level in the culture media was analyzed by using Nitrate/Nitrite Colorimetric Assay kit (Cayman Chem. Co.). Assays were performed according to the manufacturer’s protocol. Nitrate standard provided in the kit was used to construct the standard curve. Briefly, 100 μl of the medium supernatant was mixed with 100 μl of Griess reagent, and the absorbance was measured at 540/550 nm using VersaMax™ micro-plate reader (Molecular Device, USA).

### Determinations of prostaglandin E_2_ concentrations

The nitrite level in the culture media was analyzed by using PGE_2_ assay kit (R&D system, Parameter™). Assays were performed according to the manufacturer’s protocol. PGE_2_ standard and RD5-39 provided in the kit was used to construct the standards curve. Briefly, 100 μl of the medium supernatant was mixed with 50 μl of primary antibody solution and PGE_2_ conjugate. After 2 h incubation in room temperature with shaker, 96 well was washed 400 μl 1X washing buffer. Color reagent 200 μl was added, the stop solution 50 μl was mixed after 30 min. The absorbance was measured at 450/570 nm using VersaMax™ micro-plate reader (Molecular Device, USA).

### Extraction of nuclear protein

Nuclear protein extracts were prepared form RAW 264.7 macrophages using nuclear extract kit (abcam. USA). Nuclear extractions were obtained according to the manufacturer’s protocol. Briefly, the cells were washed in 1 ml of ice-cold PBS in the presence of Phosphatase inhibitors to limit further protein modifications then centrifuged at 500 rpm for 5 min in pre-cooled at 4°C. Gently re-suspend cells in 250 μl of ice-cold 1X Hypotonic Buffer. Transfer to a micro-centrifuge tube then incubate for 15 min on ice. Add 10 μl Detergent and vortex 10 seconds at highest setting. Centrifuge suspension for 30 seconds at 14,000 rpm in micro-centrifuge pre-cooled at 4°C. Re-suspend nuclear pellet in 25 μl Complete Lysis Buffer (10 mM DTT 2.5 μl, Lysis Buffer AM1 22.25 μl, Protease Inhibitor Cocktail 0.25 μl) then vortex 10 seconds at highest setting. Incubate suspension for 30 min on ice on a rocking platform set at 150 rpm. Vortex 30 seconds then centrifuge for 10 min at 14,000 rpm in a micro-centrifuge pre-cooled at 4°C. Transfer supernatant (nuclear fraction) into a pre-chilled micro-centrifuge tube.

### Western blot analysis

Cellular proteins were extracted from PY treated RAW 264.7 cells in the presence or absence of LPS (10 μg/ml) for 18 h. Cells were collected by centrifugation and washed once with ice-colded phosphate buffered saline (PBS). The washed cell pellets were collected by centrifugation and washed once with phosphate-buffered saline (PBS). The washed cell pellets were re-suspended in PRO-PREP™ protein extraction solution (Intron Biotechnology, Seoul, Korea) and incubated for 20 min at 4°C. Cell debris was removed by micro-centrifugation, followed by quick freezing of the supernatants. The protein concentration was determined using the Bio-Rad protein assay reagent according to the manufacturer’s instructions. Cellular protein from treated and untreated cell extracts was electro-blotted onto a PVDF membrane following separation on a 10–12% SDS–polyacrylamide gel electrophoresis. The immune blot was incubated for 1 h with blocking solution (5% skim milk) at room temperature. After the membrane was lightly washed with TBST, it was incubated overnight at 4°C with 1st Ab (1:1000 dilution in TBST) and then washed three times with TBST. Anti-rabbit IgG, the secondary Ab (1:2000 dilution in the blocking solution), was added, followed by incubation for 1 h at RT, then blots were again washed three times with TBST and reacted to BCIP-NBT solution (Nakanai tesque, Japan). iNOS, COX-2, p50 and b-actin monoclonal antibodies and the peroxidase-conjugated secondary antibody were purchased from Santa Cruz Biotechnology, Inc. (CA, USA). IκB-α antibody was purchased from Cell Signaling Technology, Inc. (MA, USA).

### HPLC analysis

One miligram of dried methanol extract and Ethyl acetate fraction of PY was dissolved in 2 ml of 50% acetonitrile (Duksan Chemical, Korea) and ultra-pure distilled water and filtered through 0.45 μm syringe filter (PVDF, Advantec., Japan). The HPLC apparatus was a Gilson System equipped with a 234 Auto-sampler, a UV/VIS-155 detector and a 321 HPLC Pump (Gilson, U.S.A.). Luna C_18_ reversed-phase column with 5- μ particles and 4.60 × 250 mm (Phenomenex, USA) was operated. The chromatographic separation was carried out using gradient solvents system with acetonitrile (Duksan Chemical, Korea) and water (with 0.01% formic acid) within 30 min (0 ~ 20 min: acetonitrile 20% → 40%, 20 ~ 30 min: acetonitrile 40%). The column eluent was monitored at UV 245 nm and then all solvents were degassed with a micro membrane filter (PTFE, Advantec., Japan). The chromatography was performed at room temperature with a flow-rate of 1.0 ml/min, and 10 μl volume was analyzed. PY was characterized based on the content of prunetin 5-O- β –glucopyranoside, 4′-hydroxy-tectochrysin-5-O-β-glucopyranoside and Genistein 7-O- β –glucopyranoside.

### Statistical analysis

The results were expressed as mean ± S.E. for the number of experiments. Statistical significances were compared between each treated group and analyzed by the Student’s *t*-test. Each experiment was repeated at least three times and yielded comparable results. *P*-value of < 0.05(*), p < 0.01(**), p < 0.001(***) was considered statistically significant.

## Results

### Effect of PY on cell viability in RAW 264.7 cells

**Figure 1:**
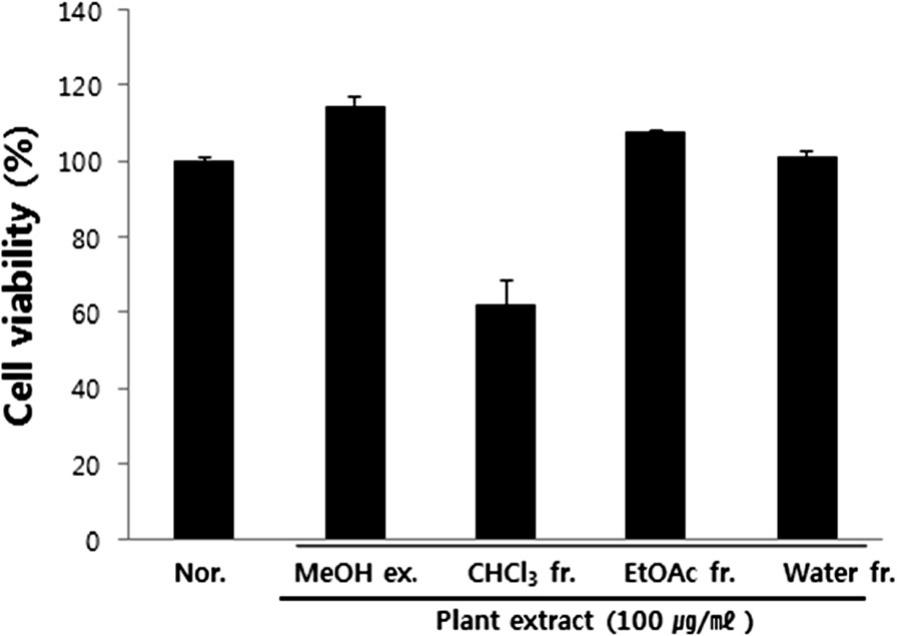
**Effect of PY on cell viability in RAW 264.7 cells.** The cells were incubated 24 h and then pretreated with 100 μg/ml different PY extracts in 1 hour. Cell viability (5 × 10^5^ cells/ml) was determined by MTS assay in the presence or absence of LPS (10 μg/ml) for 24 h. Data represent the mean ± SE of at least three independent experiments (*p < 0.05, **p < 0.01, ***p < 0.001).

The viability of cultured RAW 264.7 cells was examined by the MTS assay to test whether PY affects RAW 264.7 macrophages. Cell numbers after 24 h of incubation were not affected by 4 different extracts of PY at 100 μg/ml in the presence of LPS (10 μg/ml), except for the activity of the chloroform fraction. Chloroform extracts at 100 μg/ml concentration were therefore excluded from the whole experiment (Figure 1).

### Effect of PY on NO production

**Figure 2:**
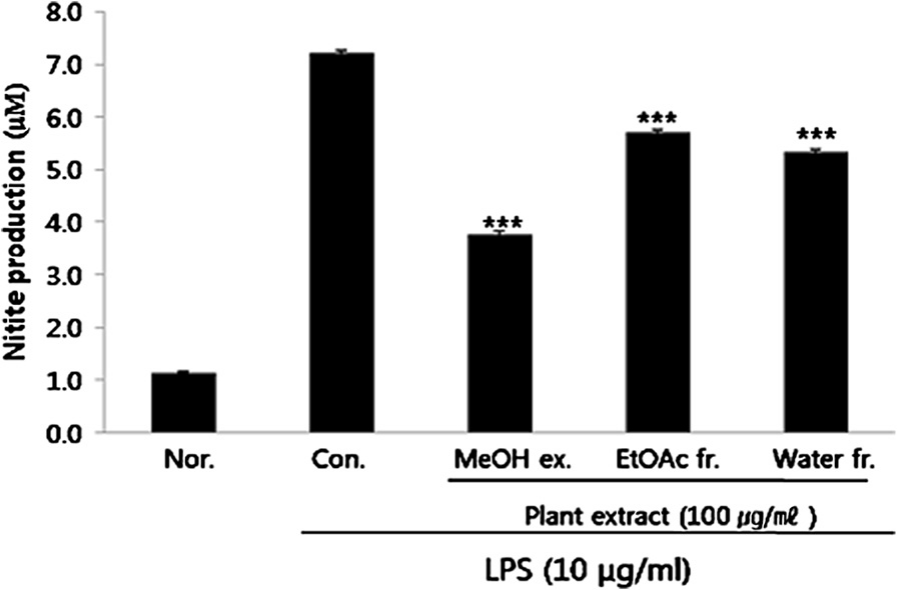
**Effect of PY extracts on lipopolysaccharide (LPS)-induced NO production in RAW 264.7 cells.** The cells were incubated 24 h and then pretreated with 100 μg/ml different PY extracts in 1 h. NO production was measured by Nitrite Colorimetric Assay kit as described in the methods in the presence or absence of LPS (10 μg/ml) for 18 h. Control (Con.) cells were incubated vehicle alone. Data represent the mean ± SE of at least three independent experiments (*p < 0.05, **p < 0.01, ***p < 0.001).

To investigate the effect of PY on NO production, accumulation of nitrite, a stable oxidized product of NO, in the culture media was measured. NO production was examined in RAW 264.7 cells stimulated by LPS in the presence or absence of MeOH, EtOAc, or aqueous (water) extracts of PY for 18 h. Media of LPS (10 μg/ml)-stimulated cells contained significantly increased nitrite levels, unlike that in the controls. The amount of nitrite increased from 1.1 ± 0.0 μM to 7.2 ± 0.1 μM. MeOH, EtOAc, or aqueous extracts of PY at a concentration of 100 μg/ml each inhibited nitrite accumulation to 47.7%, 20.9%, and 25.9%, respectively (Figure 2).

### Effect of PY on iNOS protein expression

**Figure 3:**
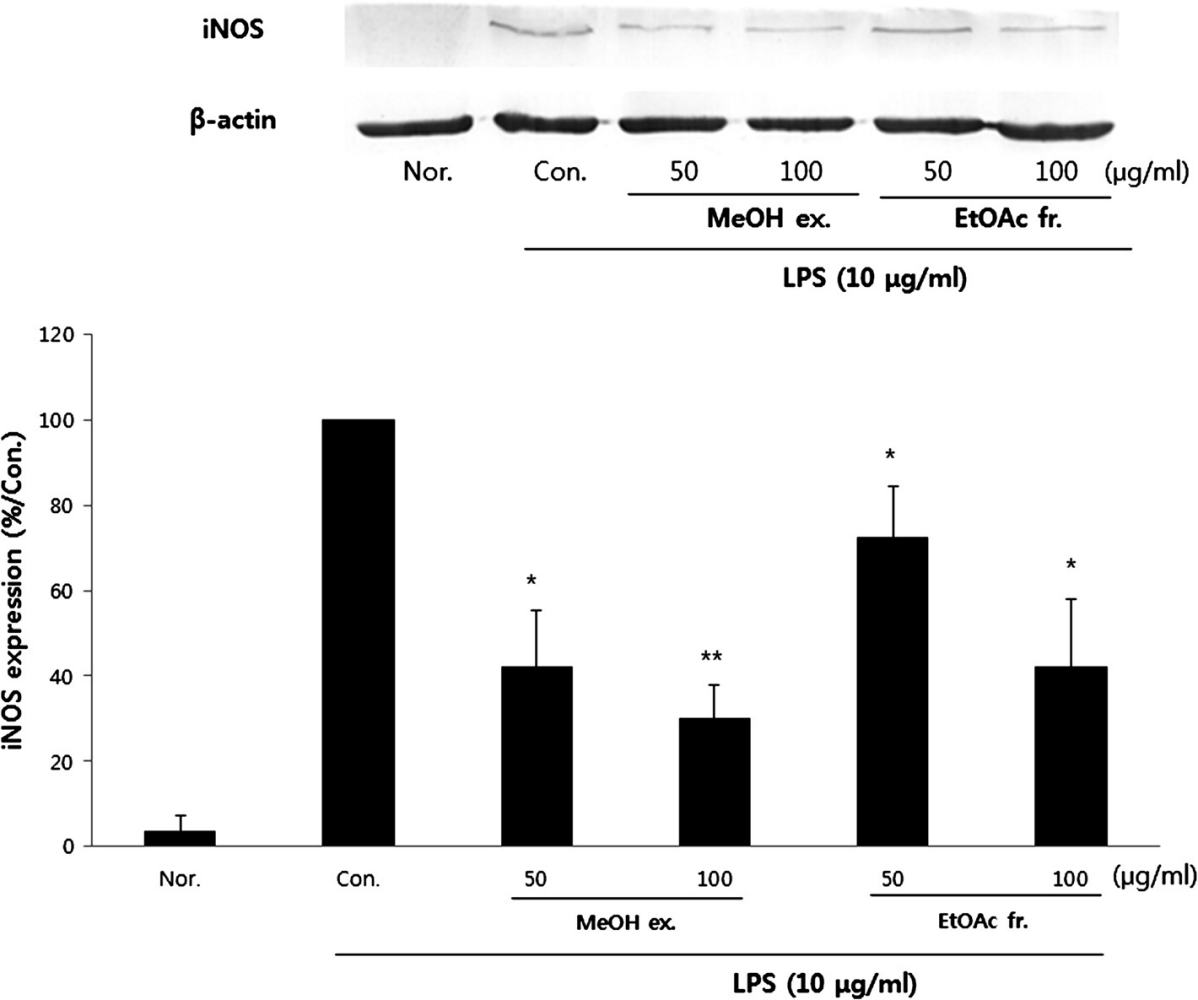
**Effect of PY extracts on lipopolysaccharide (LPS)-induced iNOS expression in RAW 264.7 cells.** The cells were incubated 24 h and then pretreated with 100 μg/ml different PY extracts (1 h) in the presence or absence of LPS (10 μg/ml) for 18 h. Protein (100 μg) from each sample was resolved 10% SDS-PAGE, and western blotting performed. B-actin was used as a control. Typical result from three independent experiments is shown (*p < 0.05, **p < 0.01, ***p < 0.001).

In order to determine the mechanisms by which PY reduces LPS-induced NO production, iNOS protein expression was measured in RAW 264.7 cells exposed to LPS (10 μg/ml) for 18 h. MeOH and Ethyl acetate extracts of PY inhibited the induction of iNOS by LPS (10 μg/ml) (Figure 3).

### Effect of PY on PGE_2_ production

**Figure 4:**
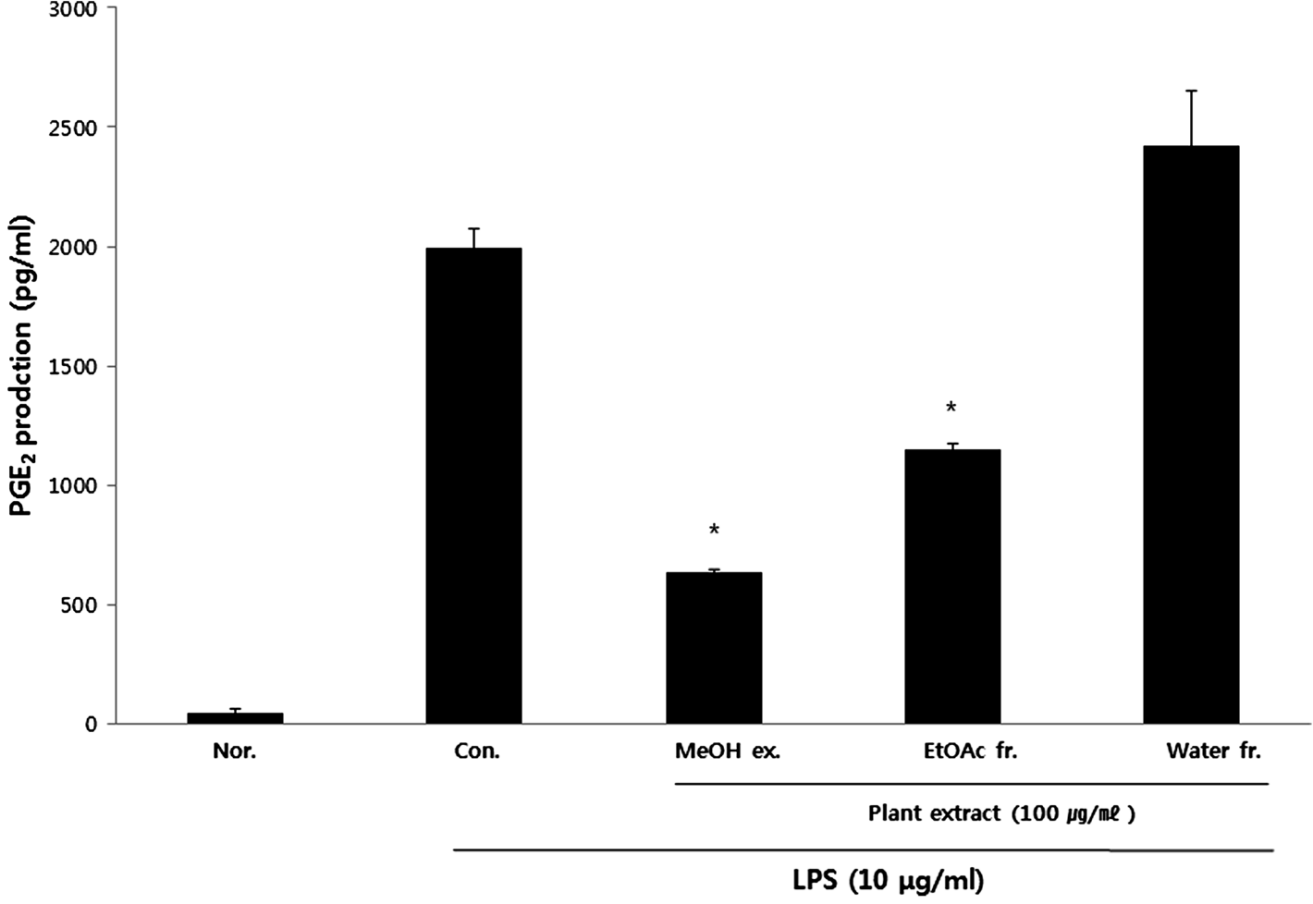
**Effect of PY extracts on lipopolysaccharide (LPS)-induced PGE**_**2 **_**production in RAW 264.7 cells.** The cells were incubated 24 h and then pretreated with 100 μg/ml different PY extracts in 1 h. NO production was measured by PGE_2_ assay kit as described in the methods in the presence or absence of LPS (10 μg/ml) for 18 h. Control (Con.) cells were incubated vehicle alone. Data represent the mean ± SE of at least three independent experiments (*p < 0.05, **p < 0.01, ***p < 0.001).

To investigate the effect of PY on PGE_2_ production, accumulation of PGE_2_ was measured. PGE_2_ accumulation was examined in RAW 264.7 cells stimulated with LPS for 18 h in the presence or absence of MeOH, EtOAc, or aqueous extracts of PY. LPS (10 μg/mL)-stimulated cells produced significantly higher PGE_2_ levels (1994.4 ± 81.8 pg/mL in culture media) compared to controls (47.0 ± 17.2 pg/mL). The inhibition rates of MeOH, EtOAc extracts were 68.0%, 42.3% in 100 μg/ml, each sample concentration (Figure 4).

### Effect of PY on COX-2 protein expression

**Figure 5:**
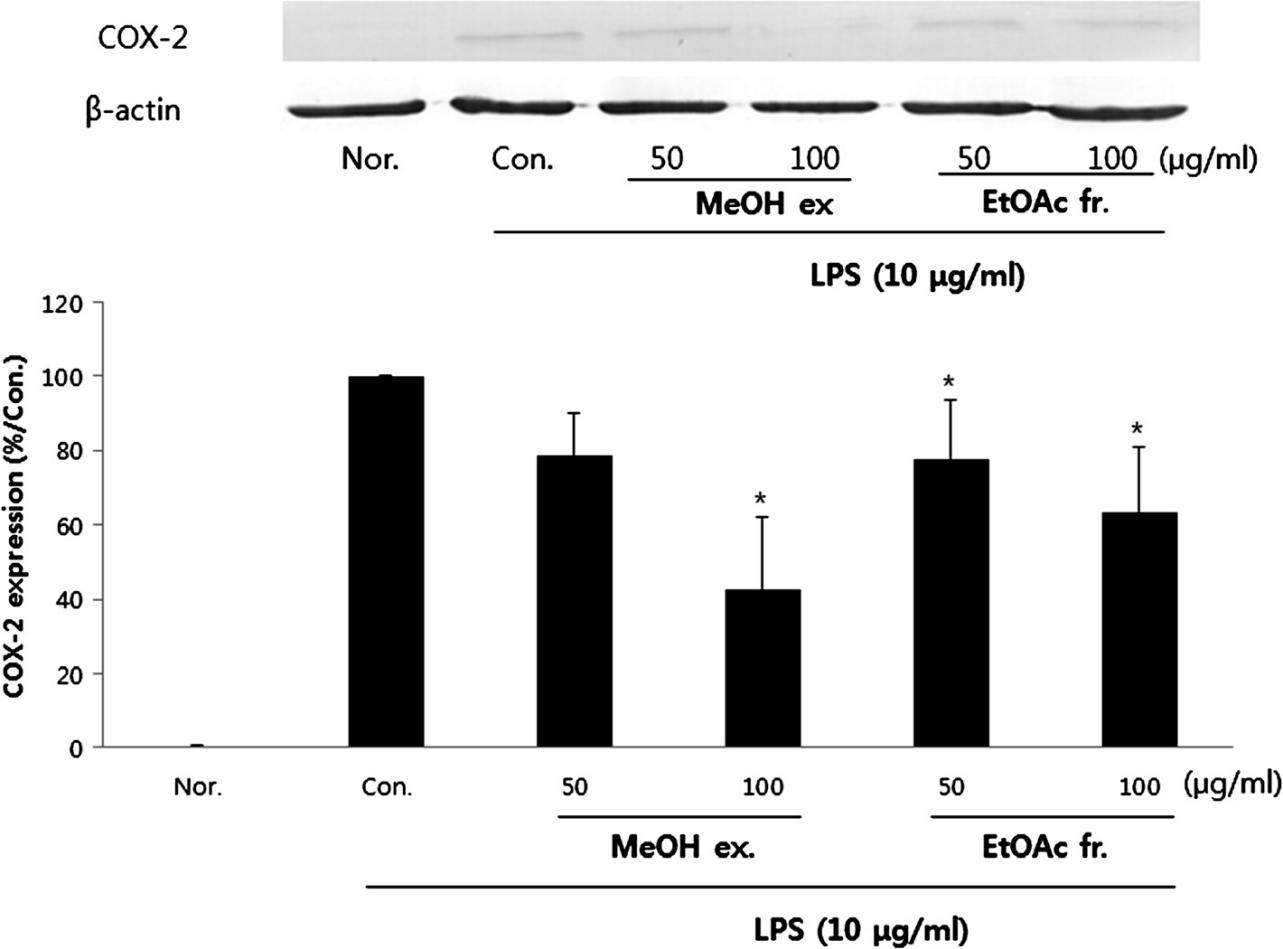
**Effect of PY extracts on lipopolysaccharide (LPS)-induced COX-2 expression in RAW 264.7 cells.** The cells were incubated 24 h and then pretreated with 100 μg/ml different PY extracts (1 h) in the presence or absence of LPS (10 μg/ml) for 18 h. Protein (100 μg) from each sample was resolved 10% SDS-PAGE, and western blotting performed. B-actin was used as a control. Typical result from three independent experiments is shown (*p < 0.05, **p < 0.01, ***p < 0.001).

In order to determine the mechanisms by which PY reduces LPS-induced PGE2 production, the COX-2 protein expression was measured in RAW 264.7 cells exposed to LPS (10 μg/ml) for 18 h. MeOH and Ethyl acetate extracts of PY inhibited the induction of COX-2 protein by LPS (10 μg/ml) (Figure 5).

### Effect of PY on NF-κB translocation

**Figure 6:**
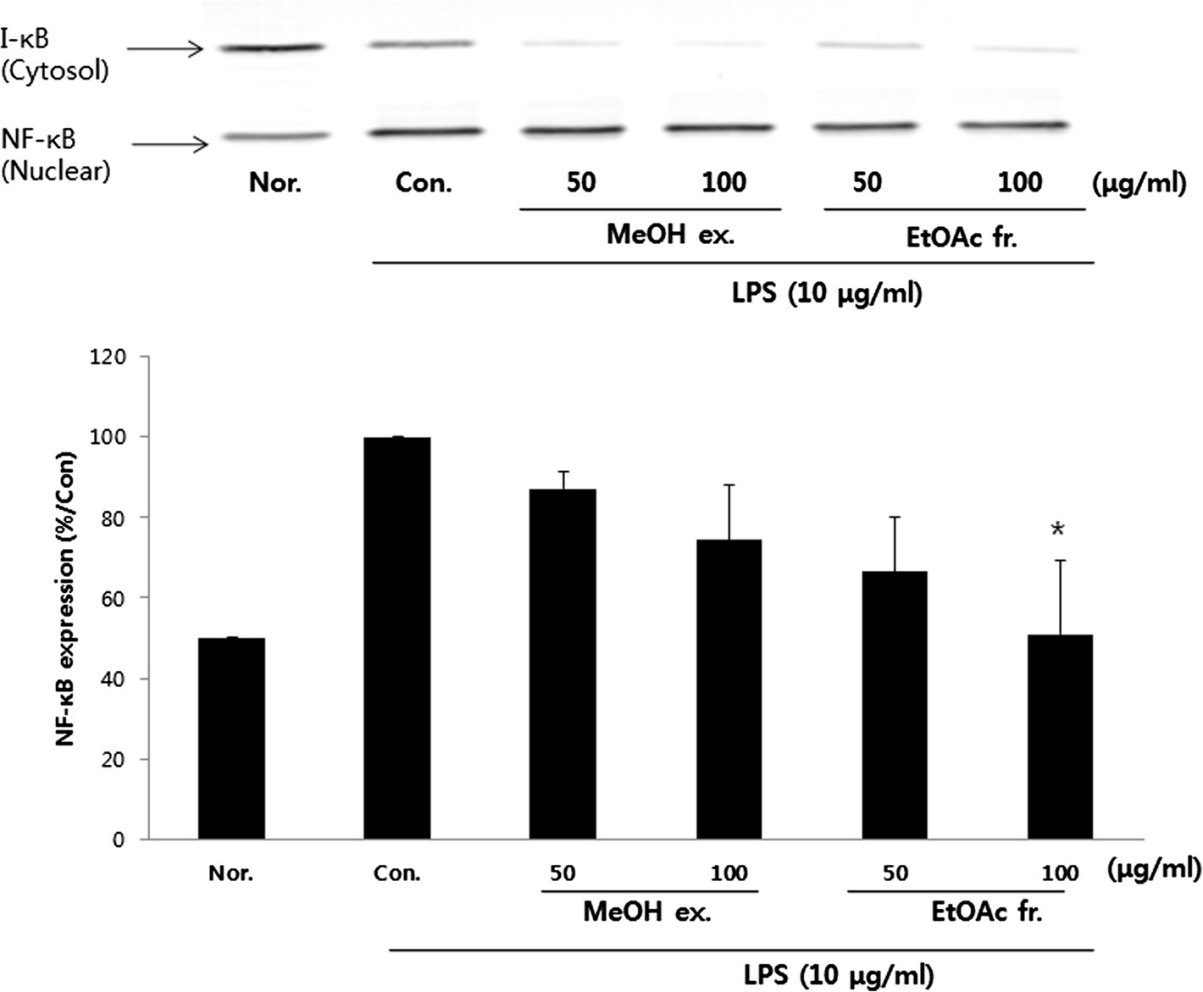
**Effect of PY extracts on lipopolysaccharide (LPS)-induced NF-κB expression in RAW 264.7 cells.** The cells were incubated 24 h and then pretreated with 100 μg/ml different PY extracts (1 h) in the presence or absence of LPS (10 μg/ml) for 18 h. Nuclear protein was obtained as described in the methods Nuclear protein was resolved 12% SDS-PAGE, and western blotting performed. Typical result from two independent experiments is shown (*p < 0.05, **p < 0.01, ***p < 0.001).

Given that the NF-κB pathway is one of the major signalling pathways leading to the activation of iNOS and COX genes, we evaluated the effect of PY on the nuclear distribution of the p50 NF-κB protein following PY treatment. LPS markedly induced the translocation of p50 to the nucleus, and PY pretreatment significantly suppressed this translocation (Figure 6). We also examined whether PY inhibited the phosphorylation and subsequent degradation of the protein, inhibitor of (NF) κB, (IκB-α). PY pretreatment significantly reduced LPS-induced IκB-α phosphorylation and prevented IκB-α degradation (Figure 6).

### Comparing the constituents of methanol and ethyl acetate extracts by HPLC

**Figure 7:**
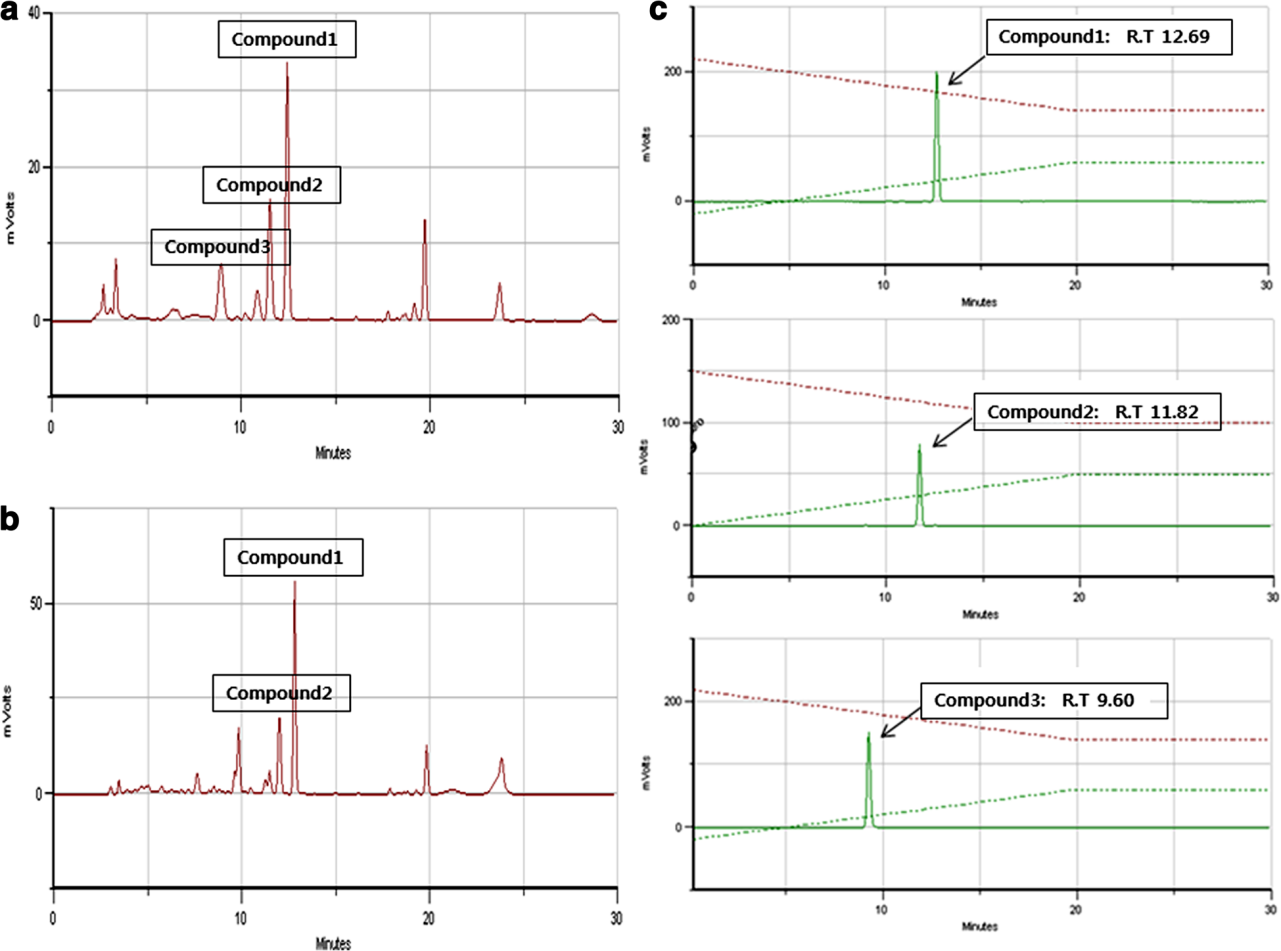
**HPLC analysis of the PY and its Ethyl acetate extracts. Standard solution of PY was prepared by dissolving in pure methanol (HPLC grade, 2 g/100 ml).** The injection volume was 10 μl and the detection was made at 254 nm. (**a**) The chromatography of MeOH extract. (**b**) The chromatography of Ethyl acetate extract. (**c**) The chromatography of standard compound. Typical result from three independent experiments is shown (*p < 0.05, **p < 0.01, ***p < 0.001).

In an effort to compare and characterize the main constituents of the methanol and Ethyl acetate extracts of PY, they were analysed by HPLC method. A chromatogram of the MeOH extract of PY is shown in Figure 7(a) and that of the ethyl acetate extract of PY is shown in Figure 7(b). A comparison of the chromatograms of both the extracts shows that they have similar elution profiles and retention time for the major peaks. The PY extracts were characterized based on the content of prunetin 5-O- β –glucopyranoside, 4′-hydroxy-tectochrysin-5-O-β-glucopyranoside, and genistein 7-O- β –glucopyranoside. The retention times of prunetin 5-O- β –glucopyranoside, 4′-hydroxy-tectochrysin-5-O-β-glucopyranoside, and Genistein 7-O- β –glucopyranoside in PY extracts were 12.69, 11.82, and 9.60 min, respectively (Figure 7).

## Discussion

The present study was undertaken to elucidate the pharmacological and biological effects of PY on the production of inflammatory mediators in macrophages. The results indicate that methanol and ethyl acetate extracts of PY (50, 100 μg/ml) were effective inhibitors of LPS-induced NO, and PGE2 production in RAW 264.7 cells. The data demonstrated that these inhibitory effects were accompanied by a decrease in the expression levels of iNOS and COX-2 expression in RAW 264.7 macrophages mediated by the methanol and ethyl acetate extracts of PY (50, 100 μg/ml). This suppression was in turn related to the decrease of p50NF-κB nuclear translocation.

Inflammatory mediators such as NO and pro-inflammatory cytokines are involved in host defence mechanisms, and their overproduction contributes to the pathogenesis of several diseases including periodontitis, bacterial sepsis, rheumatoid arthritis, chronic inflammation, and hepatitis [16,20-22]. During infection and inflammation, the increased production of NO has been shown to cause mutations and DNA damage [23]. Therefore, pharmacological interference of NO production has been speculated to be useful in alleviating numerous disease states that are mediated by increased and/or protracted activation of macrophages. The data of the present study indicates that PY reduced NO production in RAW 26.7 cells.

Prostaglandins also play a major role as mediators of the inflammatory response. Cyclooxygenase (COX) is an enzyme that converts arachidonic acid to prostaglandins [24]. Like NOS, 2 isoforms of COX have been found: COX-1 and COX-2. COX-1 is expressed constitutively in most tissues and is responsible for the homeostatic production of prostaglandins. In contrast, COX-2 is induced by several stimuli, including growth factors, mitogens, cytokines, and tumour promoters. It is responsible for the production of large amounts of pro-inflammatory prostaglandins at the site of inflammation, and its uncontrolled activity is thought to play an important role in the pathogenesis of many chronic inflammatory diseases [25-27]. Our data suggests that PY treatment suppressed LPS-induced expression of COX-2, iNOS, and PGE_2_ in RAW 264.7 cells.

NF-κB is composed mainly of 2 proteins, p50 and p65 [28]. In stimulated cells, NF-κB is present in the cytoplasm and is bound to the inhibitory protein I-κB. Exposure of cells to various NF-κB activators such as LPS or TNF-α, results in phosphorylation and degradation of the inhibitory protein I-κB, leading to the release of NF-κB from I-κB and its translocation into the nucleus. This study demonstrates the inhibition of LPS-induced I-κB degradation in the cytosol and a suppression of LPS-induced activation of NF-κB in the nucleus due to the action of methanol and ethyl acetate extracts of PY.

Genistein (4′, 5, 7-trihydroxyisoflavone) is a naturally occurring flavone and the major isoflavone in soybean. Genistein has been reported to have numerous anti-oxidative and anti-cancer effects and is known to inhibit tyrosine-specific protein kinases. Recent studies have demonstrated that genistein suppresses LPS-induced inflammatory response by inhibiting NF-κB following AMP kinase activation in RAW 264.7 Macrophages [29-34]. We found that extracts of PY contained Genistein 7-O- β –glucopyranoside, prunetin 5-O- β –glucopyranoside and 4′-hydroxy-tectochrysin-5-O-β-glucopyranoside.

## Conclusion

In conclusion, we have demonstrated that methanol and ethyl acetate extracts of PY inhibit LPS-induced NO, PGE_2_ production, as well as iNOS and COX-2 expression in macrophages. This anti-inflammatory effect occurred via the suppression of the p50 NF-κB nuclear translocation in LPS-induced RAW 264.7 cells and subsequent downregulation of iNOS and COX-2 expression. These data therefore indicate the presence of a novel mechanism of action underlying the apparent anti-inflammatory efficacy of this traditional herbal medicine.

## Competing interests

The authors declare that they have no competing interests.

## Authors’ contributions

JL and GY designed the study, conducted the experiments, and wrote the manuscript. KL performed the experiments, analyzed the data. ML designed the study and wrote the manuscript. JE and IH helped to perform the experiment. HC provided the initial idea, instructed the study, and wrote the manuscript. All authors read and approved of the final manuscript.

## Pre-publication history

The pre-publication history for this paper can be accessed here:

http://www.biomedcentral.com/1472-6882/13/92/prepub
